# Characterising the intensity of insecticide resistance: A novel framework for analysis of intensity bioassay data

**DOI:** 10.1016/j.crpvbd.2023.100125

**Published:** 2023-06-16

**Authors:** Mara D. Kont, Ben Lambert, Antoine Sanou, Jessica Williams, Hilary Ranson, Geraldine M. Foster, Rosemary S. Lees, Thomas S. Churcher

**Affiliations:** a MRC Centre for Global Infectious Disease Analysis, Department of Infectious Disease Epidemiology, Imperial College London, Norfolk Place, London, W2 1PG, UK; b Department of Mathematics and Statistics, University of Exeter, Exeter, EX4 4QJ, UK; c Centre National de Recherche et de Formation sur le Paludisme, Ouagadougou, Burkina Faso; d Department of Vector Biology, Liverpool School of Tropical Medicine, Liverpool, L3 5QA, UK

**Keywords:** Insecticide resistance, Phenotypic resistance monitoring, Bayesian dose-response modelling, Temporal analysis

## Abstract

Insecticide resistance is a growing problem that risks harming the progress made by vector control tools in reducing the malaria burden globally. New methods for quantifying the extent of resistance in wild populations are urgently needed to guide deployment of interventions to improve disease control. Intensity bioassays measure mosquito mortality at a range of insecticide doses and characterise phenotypic resistance in regions where resistance is already detected. These data are increasingly being collected but tend to exhibit high measurement error and there is a lack of formal guidelines on how they should be analysed or compared. This paper introduces a novel Bayesian framework for analysing intensity bioassay data, which uses a flexible statistical model able to capture a wide variety of relationships between mortality and insecticide dose. By accounting for background mortality of mosquitoes, our approach minimises the impact of this source of measurement noise resulting in more precise quantification of resistance. It outputs a range of metrics for describing the intensity and variability in resistance within the sample and quantifies the level of measurement error in the assay. The functionality is illustrated with data from laboratory-reared mosquitoes to show how the lethal dose varies within and between different strains. The framework can also be used to formally test hypotheses by explicitly considering the high heterogeneity seen in these types of data in field samples. Here we show that the intensity of resistance (as measured by the median lethal dose (LC_50_) of insecticide) increases over 7 years in mosquitoes from one village in Burkina Faso but remains constant in another. This work showcases the benefits of statistically rigorous analysis of insecticide bioassay data and highlights the additional information available from this and other dose-response data.

## Highlights

• Modelling intensity assay dose-response better characterises insecticide resistance.
• Bayesian model of intensity assays show temporal changes in insecticide resistance.
• Mosquito population resistance heterogeneity can be defined from intensity assays.

## Graphical abstract

**Figure:**
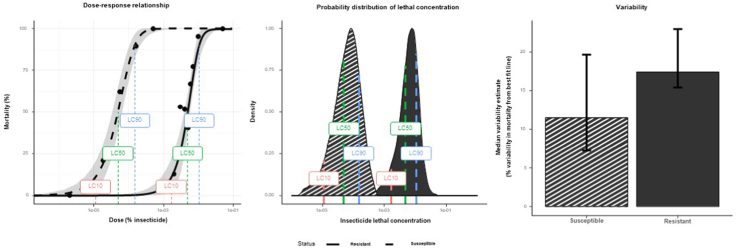

## 1 Introduction

In the fight against malaria, vector control tools have been the most impactful and cost-efficient interventions deployed. Insecticide-treated nets (ITNs) and indoor residual spraying (IRS) are estimated to have contributed to a decrease of 517 million clinical malaria cases between 2000 and 2015 (Bhatt et al., 2015). However, due to continued selection pressure imposed by the use of insecticides in both public health and agriculture, insecticide resistance in disease vectors has grown and spread (WHO, 2021). At this time, resistance to all insecticides currently in use has been reported in various *Anopheles* vectors across the globe (WHO, 2019; Zoh et al., 2021). Whilst operational failure due to insecticide resistance has been demonstrated in the agricultural sector (Kranthi, 2005; Alyokhin et al., 2015), understanding this relationship at the public health level has been more complex. Early detection of potential sub-optimal responses to control tools is essential for good insecticide resistance management, but there remains debate as to whether resistance to pyrethroid insecticides used on ITNs will have a significant operational impact in the control of malaria (Thomas and Read, 2016; Kleinschmidt et al., 2018; Lindsay et al., 2021; WHO, 2022). A better characterisation of resistance is needed before epidemiological impacts can be explored, and before it is potentially too late, to mitigate its public health impact.

The processes governing insecticide resistance in malaria vectors are still not fully understood, with new mechanisms continuously being discovered (Ingham et al., 2020). A variety of genetic markers have been identified which predict varying degrees of resistance to pyrethroids (Ingham et al., 2018; Weetman et al., 2018; Adolfi et al., 2019; Enayati et al., 2020; Black et al., 2021). New mutations are still being linked with resistance (Quiñones et al., 2015; Ingham et al., 2018) and other, more nuanced, mechanisms, such as cuticular changes in mosquitoes, are still being identified (Githeko et al., 1996; Sokhna et al., 2013; Ingham et al., 2020; Lissenden et al., 2021; Sanou et al., 2021). Whilst understanding the different mechanisms at play in resistant mosquitoes is vital in combatting resistance, monitoring phenotypic implications of this resistance is currently most useful from a programmatic perspective (WHO, 2022). Discriminating dose bioassays (DDBs, also known as susceptibility assays) are a quick and cheap method and are widely used across the world (WHO, 2016). This assay involves exposing mosquitoes captured in the field as adults or larvae, then reared to adulthood in the laboratory, to a defined concentration of insecticide for a set amount of time and recording mosquito mortality after 24–72 ​h (WHO, 2016, 2022). This concentration should be able to discriminate between susceptible and resistant mosquitoes: if less than 98% of the mosquitoes tested in the assay die, that sample is considered resistant (WHO, 2016). However, these assays lack sensitivity, as the resistant alleles would need to be well established to be observed phenotypically (Kranthi, 2005; Riveron et al., 2018), but also as they fail to differentiate between mosquito populations with moderate and high intensity resistance. Both may survive the discriminatory dose but may not be killed by the doses seen on ITNs and IRS. DDBs are also prone to measurement error, partly because of the highly variable nature of entomological samples which are affected by differences in the environment, such as temperature, humidity, time of day or larval breeding sites (Spillings et al., 2008; Glunt et al., 2011, 2018; Jones et al., 2012; Oliver and Brooke, 2014). Furthermore, spatial distribution of sampling (WHO, 2016) and the concentration set as ‘discriminating’ will also strongly determine whether resistance is detected (Halliday and Burnhaw, 1990; WHO, 2012; Lissenden et al., 2021). Whilst DDBs are good at identifying the presence of resistance and are highly scalable, they do not inform on the magnitude of the resistance nor necessarily indicate its public health impact (Lipnick et al., 1995; Venter et al., 2017). With malaria vectors in most countries in Africa exhibiting phenotypic resistance to pyrethroids, the single concentration used in DDBs, which is substantially lower than those used in vector control products (Bagi et al., 2015), may not be informative from a resistance management standpoint if there is a lack of alternative insecticides. Nevertheless, it is likely that the epidemiological benefit of different ITNs or IRS will vary according to the level of insecticide resistance (Sherrard-Smith et al., 2022), so there is a need for more sensitive measures of resistance to support the deployment of vector control or other anti-malarial interventions.

To better understand the magnitude of insecticide resistance in disease vectors, intensity bioassays (IBs, also referred to as dose-response bioassays) were recommended for use by the World Health Organization (WHO) in 2016 (WHO, 2016). These assays build on DDBs by adding further replicates at different (higher) insecticide concentrations using mosquitoes from the same sampling population (Bagi et al., 2015). Based on the percentage of mosquitoes dying at each concentration (i.e. 1× ​, 5× ​and 10× ​the discriminating dose), the WHO will then classify the sample as having low, moderate or high resistance, respectively (WHO, 2016). Beyond quantifying the intensity of resistance, combining the replicate data across different concentrations in IBs can yield more precise estimates compared to single DDBs, as metrics can be estimated from the dose-response relationship rather than mortality at a set dose (WHO, 2016; Burgess et al., 2020). How best to analyse IB data in a public health context, and what their results mean practically for operational use is still uncertain (WHO, 2022). Fitting statistical models to results from multiple assays allows the calculation of the lethal concentration of insecticide that kills 50% of a sample population (LC_50_). Different statistical techniques have been used to estimate LC_50_ (Burgess et al., 2020); however, it is unclear whether they can cope with the high variability and lack of replicates typically seen in mosquito surveillance data, are flexible enough to capture subtle differences between mosquito populations, or properly account for different levels of background mortality. This background mortality is measured in mosquitoes not exposed to insecticide, and generally, if mortality is greater than 5% then DDBs and IBs mortality estimates should be adjusted for using the Abbott formula (Abbott, 1925). If background mortality is too high (> 20%), then assays are generally repeated (WHO, 2022). Though repetition should always be encouraged, removing data above an arbitrary threshold risks biasing results and complicating analyses. This will particularly be the case for novel insecticides with slower acting neurotoxic insecticides such as chlorfenapyr which are assessed over longer periods of time (up to 72 ​h post-exposure). As their use increases, there is a need to refine the statistical analyses of these IBs, taking into account background mortality and enabling rigorous evaluation of different hypotheses, for example determining whether the intensity of resistance varies spatially or temporally.

Here, a novel and comprehensive framework for analysis of IB mortality data is introduced. The functionality is illustrated using both laboratory- and field-generated data, providing a suite of different metrics to characterise insecticide resistance and introducing statistical methods to allow hypothesis testing.

## 2 Methods

### 2.1 Datasets

IB mortality data were collated from laboratory and field experiments. The laboratory data were generated at the Liverpool School of Tropical Medicine’s Insect Testing Establishment (LITE) and are described in more detail in Williams et al. (2019). Briefly, five laboratory mosquito colonies were exposed to increasing concentrations of the pyrethroid insecticide permethrin *via* tarsal exposure by coating a glass plate with insecticide, to allow identification of cuticular resistance mechanisms (Williams et al., 2019; Choo et al., 2000). Mortality was recorded at 24 ​h. One of the colonies (Kisumu) was susceptible to pyrethroids and the other four showed differing levels and mechanisms of pyrethroid resistance (Williams et al., 2019), with a summary overview of each strain provided in Table 1.

**Table 1:** Summary of strains and resistance levels in the laboratory dataset.

Species	Strain	Insecticide resistance	Mechanism of resistance
*Anopheles gambiae* (*s.s.*)	Kisumu	Susceptible	None
*An. coluzzii*	VK7 2014	Pyrethroids; DDT (organochloride); carbamates	Target-site mutation
	Banfora	Pyrethroids; organochlorides	Target-site mutation
			Metabolic
*An. funestus* (*s.s.*)	FUMOZ-R	Pyrethroids	Metabolic
*An. gambiae* (*s.s.*) (98%); *An. gambiae* (*s.s.*) *- An. coluzzii* hybrid (2%)	Tiassalé 13	Pyrethroids; organochlorides; carbamates	Target-site mutation
			Metabolic

*Note*: The data were generated at the LITE insectaries and the details above are summarised from Williams et al. (2019), including possible primary mechanisms of resistance.

From the experiments, 91 individual mortality datapoints across the five mosquito strains were used for analysis.

Field data were collected as part of an insecticide resistance monitoring programme in two locations of south-west Burkina Faso, approximately 35 ​km away from one another: Tengrela and Tiefora (Sanou et al., 2021). Larvae were collected during the rainy season between May and November and reared to adulthood before being exposed to different doses of deltamethrin using the standard WHO tube bioassay (WHO, 2016; Sanou et al., 2021). In total, 641 individual mortality data points were obtained from 67 to 78 intensity bioassays across seven and six years in Tengrela and Tiefora, respectively.

### 2.2 The framework: Functional forms

#### 2.2.1 Base model

A binomial model using a five-parameter logistic function was developed to characterise and understand the dose-response relationship arising from intensity dose bioassays. The counts of dead mosquitoes, *y*_*i,*_ following exposure to insecticide or control (i.e. no insecticide) were assumed to be samples from a binomial distribution:

(1) $$y_{i}\;∼\;binomial\left(n_{i},p_{i}\right)\text{,}$$

where $n_{i}$ is the number of mosquitoes tested in assay *i*, and $0\leq p_{i}\leq 1$ represents the mean proportion of mosquitoes dying, as described by the following functional form:

(2) $$p_{i}=D+\frac{A-D}{{\left[1+e^{B∙\left(\ln\left(\sqrt{x_{i}}\right)-C\right)}\right]}^{E}}\text{.}$$

The parameters used in this logistic function are detailed in Table 2, with their effect shown in Supplementary file 1: Fig. S1, and *x*_*i*_ ​≥ ​0 represents the insecticide concentration in assay *i.* The square root of insecticide concentrations *x* was used when fitting the models to mitigate the impact of doubling concentrations along the mortality gradient, to normalise the concentration range. Parameters *A-C* and *E* were fit in a Bayesian framework, and the priors were assigned (Table 2) to allow a wide variety of possible dose-response relationships (see the prior predictive sets in Supplementary file 1: Fig. S2). Parameter *D* (Table 2) was fixed at 1 based on the assumption that mosquito mortality reaches 100% when exposed to sufficient quantity of the insecticide. Whilst this assumption may not hold in all situations, we included it here to be able to estimate concentration values beyond the data points if no values for 100% mortality were present in the data.

**Table 2:** Summarising the parameters of Equations (2), (3.1), (3.2), (3.3). Notation, description, prior values and restrictions are reported for each parameter.

Parameter	Restrictions	Function	Priors
*A*	0 ​< ​*A* < 1	Background mortality	*A ∼* N^+^(0, 0.1)
*B*	*B* ​> ​0	Slope and shape of curve	*B* ​∼ ​N^+^(5, 10)
*C*	None		*C* ​∼ ​N(0, 5)
*D*	*D* ​> ​0	Mortality (asymptote) at highest concentrations	Fixed at *D* ​= ​1
*E*	*E* ​> ​0	Asymmetry of the curve	*E* ​∼ ​N^+^(7, 10)
*F*	None	Continuous linear function of time (replacing parameter *C*)	*F* ​∼ ​N(0,5)
*G*	None		*G* ​∼ ​N(0,5)

*Notes*: Here, parameter *D* is fixed at *D* ​= ​1 to ensure that the curve eventually ends around 100% mortality at high doses. This will allow estimation of concentration values beyond the data points if no values for 100% mortality are present. Parameters *A-C* and *E* are fit for the base model (Equation (2)), whilst parameters *B, E, F* and *G* are fit in the time models (Equation (3.1), (3.2), (3.3)). For a graphical representation of each parameter function, see Supplementary Fig. S1.

#### 2.2.2 Temporal model

The *base model* (Equation (2)) can be extended to explore the association of mortality with covariates of interest. Here we investigate whether resistance has changed over time by allowing the resistance patterns to shift and/or warp according to the year of data collection through a set of three models allowing increasingly complex temporal variation in resistance.

(i) *Linear time model*: we allow shifts in the location of the steepest part of the dose-response curve (which generally determines the LC_50_) by allowing the *C* in Equation (2) to be a continuous and linear function of time *t*:

(3.1) $$p_{i}^{lin}=D+\frac{A-D}{{\left[1+e^{B∙\left(\ln\left(\sqrt{x_{i}}\right)-\left(F+G*t_{i}\right)\right)}\right]}^{E}}$$

(ii) *Individual time model*: we allow *C* to change freely by year:

(3.2) $$p_{i}^{ind}=D+\frac{A-D}{{\left[1+e^{B∙\left(\ln\left(\sqrt{x_{i}}\right)-C_{t_{i}}\right)}\right]}^{E}}$$

(iii) *Base time model*: we allow all the parameters of Equation (2) to have unique values by year (effectively, we fit Equation (2) to each year independently):

(3.3) $$p_{i}^{base\;\left(time\right)}=D_{t_{i}}+\frac{A_{t_{i}}-D_{t_{i}}}{{\left[1+e^{B_{t_{i}}∙\left(\ln\left(\sqrt{x_{i}}\right)-C_{t_{i}}\right)}\right]}^{E_{t_{i}}}}$$

The priors used for the parameters listed in Equations (3.1), (3.2), (3.3) are listed in Table 2.

### 2.3 The framework: Hypotheses and fitting process

The laboratory and field datasets were fitted separately. For laboratory data, we explored whether there was a difference in the dose-response curves at the strain level by fitting the *base model* (Equation (2)) to all laboratory data together (*no strain difference* model) and to each of the five strains individually (*strain difference* model). The two models are compared by approximate leave-one-out cross-validation (LOO-CV) (Vehtari et al., 2017; Lambert, 2018) using the R package ‘*loo*’ (Vehtari et al., 2023). This method uses the within sample data to estimate out-of-sample predictive power (where a lower value indicates that the model estimates are close to the true distribution) and is generally recommended in model comparison for Bayesian analysis (Vehtari et al., 2017). The model with the lowest generic (expected) log-predictive density (elpd, generated from the LOO-CV) is assumed to be the best-fitting. A *P-*value to determine whether the models are statistically significantly different is obtained from the *Z*-score of the difference (Vehtari et al., 2017).

For field data, we tested whether there is a change in resistance over time in each of the two villages separately. Data from each location were fit to a *fixed resistance* model (i.e. the *base model* is fit to data from all years from a given village) and the three models outlined in Section 2.2.2, which each allow temporal variation in resistance (Equations (3.1), (3.3))). The best-fitting model is selected by LOO-CV, as described above.

The models were written and fitted *via* Markov chain Monte Carlo (MCMC) using Stan (Stan Development Team, 2019) through its R interface, *rstan* (Stan Development Team, 2021) in R v4.2.1 (RStudio Team, 2020). An example model code is freely available on GitHub. All models were fitted using 4 Markov Chains for 5000 or 10,000 iterations (dependent on the time taken for the Markov chains to converge) with a 50% warm-up period. Convergence was assessed by $\hat{R}<1.01$ (Vehtari et al., 2021). Posterior predictive checks were conducted by sampling from the posterior predictive distribution to generate dose-response curves and evaluate residuals at each concentration (Gabry et al., 2019). Nested models are compared in order of complexity (as defined by the number of parameters) and more complex models are only selected if they demonstrate a significant improvement in model fit.

Model fit and variation were assessed with the *R*^*2*^ and root mean square error (RMSE) and model residuals were evaluated at each concentration.

### 2.4 Determining useful summaries of resistance

The median lethal concentration (LC_50_) indicates the concentration of an insecticide at which 50% of the mosquitoes tested die (without background mortality), with a higher LC_50_ indicating a high intensity of resistance to that insecticide. It can be calculated by rearranging Equation (2) or (3.1), (3.2), (3.3) to obtain the dose *x* at which *p*_*i*_ ​= ​0.5 (i.e. 50% of the mosquitoes die) (Supplementary file 1: Equations S3). An estimate of LC_50_ was generated for each MCMC iteration of the model parameters. From this set of estimates, the median and the 2.5th and 97.5th quantiles were used to provide the point and 95% credible interval (CI) estimates, respectively. We compared pairs of LC_50_ values by computing the probability that their posterior distributions overlapped (i.e. P[LC_50(1)_ ​> ​LC_50(2)_]) by randomly sampling LC_50_ values from each member of the pairs.

**Fig. 1:**
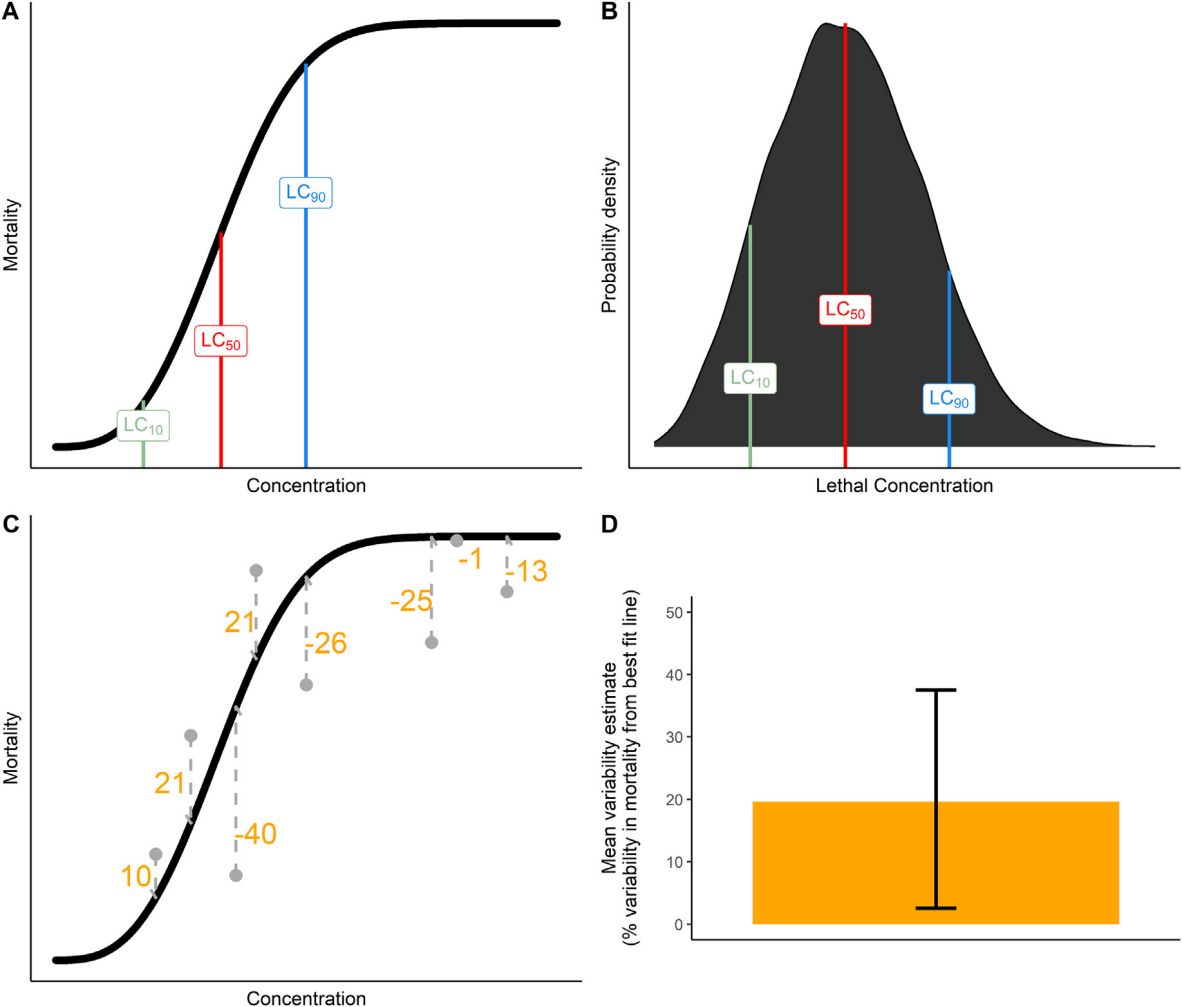
Graphical description of metrics generated by the model. Simulated data are used to generate (**A**) a dose-response curve and its respective LC_10_, LC_50_ and LC_90_ values (where the LC index describes the concentration at which that percentage of the mosquitoes die); (**B**) the probability distribution of insecticide lethal concentrations, with respective LC values from plot A mapped to it. The probability that a mosquito dies between two concentrations is the area under the curve between these concentrations; (**C**) the variability estimates (orange values) computed between the raw mortality (grey dots) and the true mortality (black curve); and (**D**) the resulting variability estimate for that bioassay. To obtain the estimate shown in **D**, the sum of the absolute distances (orange values) divided by the number of data points across all MCMC iterations (see Equation (4) and in this example, *n* ​= ​8) generates an estimate of variability.

Estimates of lethal concentrations were calculated for other levels of mortality: LC_10_ and LC_90_ (Fig. 1A). These estimates were used to measure the variability in insecticide concentration needed to kill mosquitoes within sampled populations, excluding the extreme tails of the mortality range as these will be estimated with less certainty (Halliday and Burnhaw, 1990).

The underlying probability distribution, representing the probability distribution of the lethal concentrations of the insecticides (Fig. 1B), was calculated by inverse transform sampling (Lambert, 2018).

The variability of the models was characterised by computing the mean absolute error: the absolute difference between the raw mortality data points to the fitted curve. Here, we estimate variability for each iteration of the model over the range of concentrations present in the bioassay using:

(4) $${variability}_{j}=\frac{\sum_{i=1}^{n}\left|y_{i}-y_{i,j}^{\prime }\right|}{n}$$

where *y* is the actual data point value, *y'* the simulated data point value (for each model iteration *j*), and *n* is the number of raw data points used to estimate the variability (each data point belonging to index *i*) (Fig. 1C). A summary estimate is provided for each model by taking the median and 2.5–97.5% quantiles of errors across all iterations *j* (Fig. 1D). Here, high values indicate that the observed data are different to the simulated data. The estimate is interpreted as the *percent variability in mortality from the best-fit line*.

## 3 Results

### 3.1 Laboratory data

The *strain difference model* fit to each of the five laboratory strains individually was able to better explain the variation in mortality (*R*^*2*^ ​= ​0.87–1.0, RMSE ​= ​1–10% across the five strains, Supplementary file 1: Fig. S3A) compared to the *no strain difference model* which pooled all data and estimated a single set of parameters across all strains (*R*^*2*^ ​= ​0.63, RMSE ​= ​24%, data not shown). Model comparison concluded that the *strain difference* model fit the data better (using LOO-CV, *P* ​< ​0.001).

**Fig. 2:**
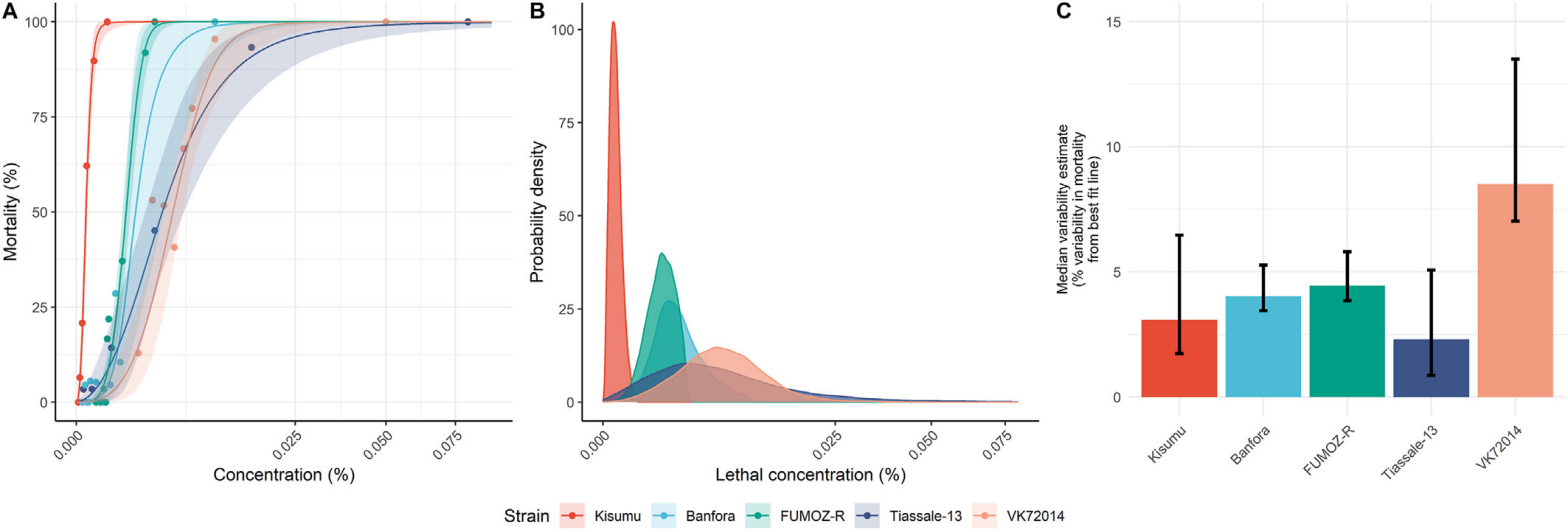
Model-derived estimates of mosquito dose-response characteristics for laboratory studies of permethrin. In panel **A**, actual (points) *versus* fitted (lines) dose-response relationships are depicted coloured according to strain; in **B**, the probability distribution of insecticide lethal concentrations is shown; and **C** depicts the estimated variability estimates. In **A**, the solid lines represent the posterior median dose-response relationship, and the shaded area around the line its 95% credible intervals. In **C**, the 95% CIs in the variability estimates are shown as error bars along with the posterior median (bars). The *Kisumu* strain (red) is susceptible and all other four strains are resistant.

When looking at individual dose-response curves from the *strain difference model* (Fig. 2A), a clear difference between the susceptible strain (Kisumu) and the four resistant strains is evident. Within the resistant strains, there was considerable variation in the shapes of the dose-response curves, resulting in different mortality densities (Fig. 2B). As expected, the susceptible strain had a substantially lower LC_50_ (4.9 ​× ​10^−5^% permethrin; 2.5–97.5% posterior quantiles, henceforth “95% CI”: 3.4 ​× ​10^−5^–6.6 ​× ​10^−5^) compared to the resistant strains, which had LC_50_ values ∼1–2 orders of magnitude higher (range: 1.3 × 10^−3^% permethrin for FUMOZ-R to 4.8 ​× ​10^−3^% permethrin for VK7 2014; Table 3). For all resistant strains, we observe a higher LC_50_ estimate compared to the susceptible strain, with P$\left[{LC}_{50}^{resistant}>{LC}_{50}^{susceptible}\right]$ ​> ​4999/5000 when random sampling across the posterior distributions. The VK7 2014 and Tiassalé 13 strains both exhibited higher intensities of resistance than either the Banfora or FUMOZ-R strains, with FUMOZ-R being distinctly less resistant than both VK7 2014 and Tiassalé 13 based on their LC_50_ values (Table 3). Examining the concentrations at which mosquitoes die (Fig. 2B), the aforementioned trends are clear. Yet the Tiassalé 13 had a much greater range of concentrations where mosquitoes typically died than the other strains (reflected in a larger difference in LC_10_ and LC_90_ values for that strain; Table 3). The model for VK7 2014 had the greatest variability (Fig. 2C), with the other four strains having considerably lower variability. But, overall, the fit of the models to the data was generally reasonable in all cases (Supplementary file 1: Fig. S3).

**Table 3:** Summarising the data and estimates for the laboratory experiments. For each laboratory strain, the number of raw data points (*N* data points) and mosquitoes (*n* mosquitoes tested) used to fit the base model is provided. Model estimates of the median lethal concentration (LC_50_) at the strain level were summarised by generating the mean across all MCMC iterations and its 95% credible intervals (CIs).

Resistance status	Strain	*N*	*n*	Concentrations tested (% permethrin)	LC_50_ (% permethrin)		Heterogeneity (% permethrin)			Background mortality (%)		Mortality variability (%)	
					Mean	95% CI	LC_10_	LC_90_	Difference	Mean	95% CI	Median	95% CI
Susceptible	Kisumu	6	196	0–0.0005	4.9 ​× ​10^−5^	3.4 ​× ​10^−5^–6.6 ​× ​10^−5^	1.1 ​× ​10^−5^	1.5 ​× ​10^−4^	1.4 ​× ​10^−4^	3	0–8	3.1	1.7–6.5
Resistant	Banfora	11	234	0–0.01	2.1 ​× ​10^−3^	1.2 ​× ​10^−3^–4 ​× ​10^−3^	8.4 ​× ​10^−4^	4.4 ​× ​10^−3^	3.5 ​× ​10^−3^	2	0*–*6	4	3.5–5.3
	FUMOZ-R	9	294	0–0.0032	1.3 ​× ​10^−3^	1.1 ​× ​10^−3^–1.5 ​× ​10^−3^	5.7 ​× ​10^−4^	2.3 ​× ​10^−3^	1.7 ​× ​10^−3^	2	0*–*6	4.4	3.8–5.8
	Tiassalé 13	8	238	0–1.97	3.9 ​× ​10^−3^	2.5 ​× ​10^−3^–5.9 ​× ​10^−3^	7.4 ​× ​10^−4^	1.5 ​× ​10^−2^	1.4 ​× ​10^−2^	4	0*–*10	2.3	0.9–5.1
	VK7 2014	8	277	0–0.05	4.8 ​× ​10^−3^	4.0 ​× ​10^−3^–5.9 ​× ​10^−3^	1.7 ​× ​10^−3^	1.1 ​× ​10^−2^	8.9 ​× ​10^−3^	9	0*–*22	8.5	7–13.5

*Notes*: Mosquito population heterogeneity is investigated by examining the range of concentrations at which 10% and 90% of the mosquitoes die (mean LC_10_ and LC_90_ of all model iterations for each strain) and the difference between these two doses. The amount of background mortality is quantified from model estimates, with the mean of all model iterations for each strain and 95% credible intervals provided above. The amount of variability in mortality is quantified from the average absolute distance of the data points to the best-fit line along the mortality axis, with the median of all model iterations for each strain and 95% credible intervals provided above.

*Abbreviations*: *N*, number of data points; *n*, number of mosquitoes tested.

Background mortality is generally relatively low, but not negligible: all bar one strain had estimates of background mortality below 5%, except for strain VK7 2014 for which it was 9% (Table 3). These results indicate the importance of accounting for background mortality when fitting IB data.

### 3.2 Field data

**Fig. 3:**
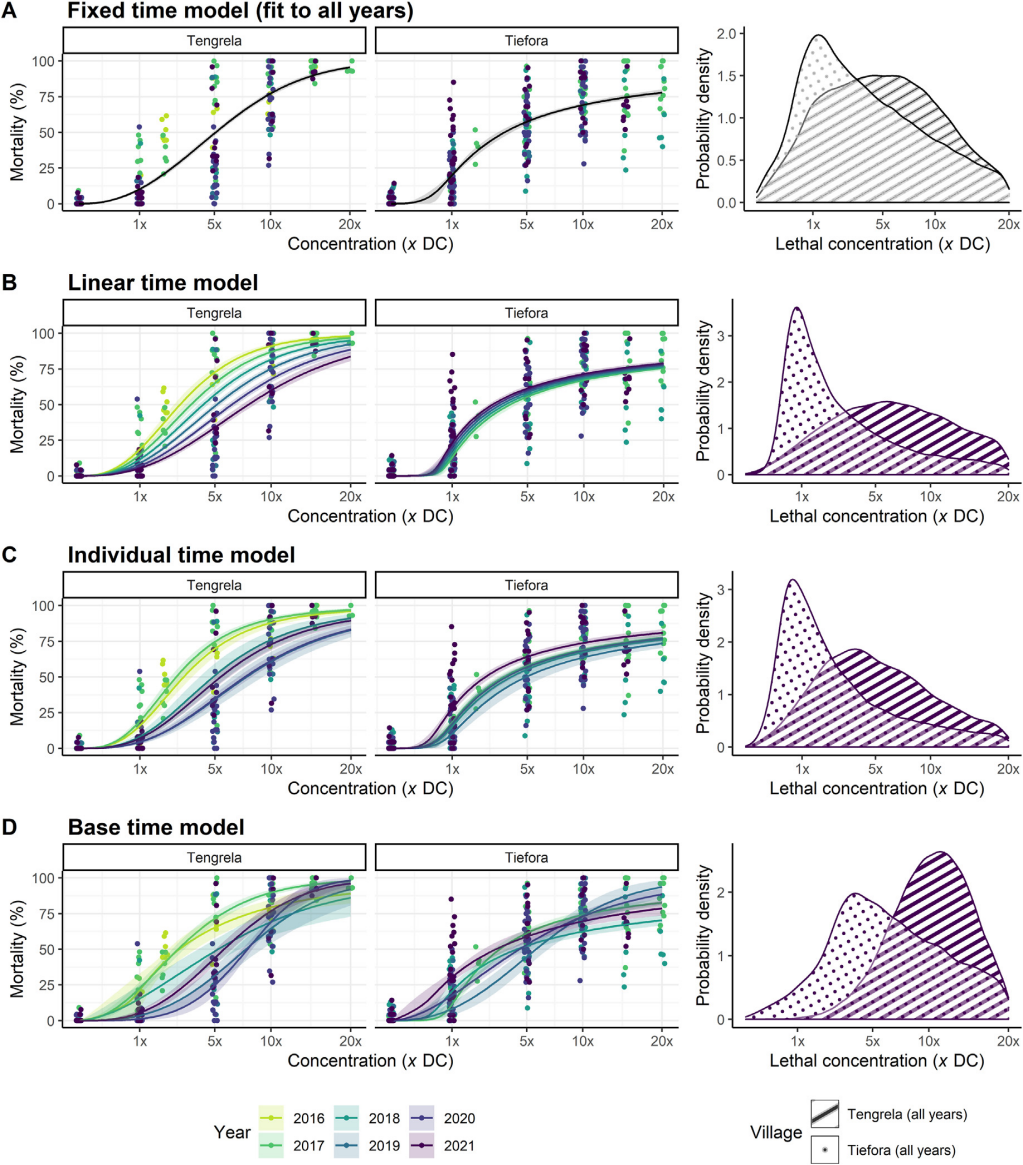
Model-derived estimates of mosquito dose-response characteristics for deltamethrin in field studies. The *base model* (Equation (2)) is fit to all years in each location in **A**, and *temporal models* depicted in Equations (3.1), (3.2), (3.3) are shown in **B**-**D**, respectively. For each panel, in the first two graphs, the actual data (points) *versus* fitted dose-response relationships (lines) are depicted for each location, coloured by year; and in the third graph, the probability distribution of insecticide lethal concentrations is shown, coloured by location. In the dose-response plots, the solid lines represent the posterior median dose-response relationship and the shaded area around the line its 2.5–97.5% posterior quantiles for each location, with *time model* parameters *F* and *G* allowed to vary linearly (panel **B**) or independently (panel **C**) by year; in panel **D**, all parameters were allowed to vary.

We fitted the field resistance data from Tengrela and Tiefora using a model which assumed a fixed dose-response relationship across all years of data collection (*fixed resistance* model, Fig. 3A) and a series of three models which allowed temporal variation in resistance profiles at increasing levels of flexibility (*resistance change* models, Fig. 3B-D). In Tengrela, there was evidence of temporal variation in resistance (LOO-CV, *P* ​< ​0.01 for all three temporal *resistance change* models *versus* the *fixed resistance* model), but not in Tiefora (Supplementary file 1: Table S1).

In Tengrela, there was a striking shift towards resistance profiles embodying substantially higher levels of resistance across a range of concentrations (Fig. 3B-D). For the *base time* model, there was a 2.8-fold increase in LC_50_ from 2016 to 2021 (Supplementary file 1: Table S2.3). For Tiefora, the change in the resistance profile over time was less uniform (with P$\left[LC_{50}^{2021}\;>\;LC_{50}^{2017}\right]$ ​= ​3582/10,000 ​= ​0.36 obtained from random sampling for the *base time* model), and, if anything, the trends appear to be towards lower resistance in later years (P$\left[LC_{50}^{2021\;}>\;\;LC_{50}^{2017}\right]$ ​< ​2/5000 for the *individual* and *linear time* models) (Supplementary file 1: Table S2).

When fitting the base model across all years, Tengrela displays higher LC_50_ than Tiefora with an LC_50_ of 5× ​the discriminating dose (DC) of deltamethrin (i.e. 0.25% deltamethrin, 95% CIs: 0.24–0.27 or 4.8–5.4× ​DC) in Tengrela compared to 3.4× ​the discriminating dose (0.17%, 95% CIs: 0.15–0.20 or 3–4× ​DC) in Tiefora (P$\left[LC_{50}^{Tengrela\;}>\;LC_{50}^{Tiefora}\right]$ ​= ​1). This indicates that over the period, mosquitoes in Tiefora are, on average, more susceptible to deltamethrin than Tengrela. This is further shown when looking at the last year data, where mosquitoes typically die at lower deltamethrin concentrations in Tiefora than Tengrela (Fig. 3B). However, there was considerable heterogeneity in the lethal concentrations in both locations for the mosquitoes surveyed (right-hand column in Fig. 3), possibly indicating large population-level heterogeneity in the level of resistance across specimens or temporal variations in species composition. Although there was considerable overlap in the distributions quantifying the lethal dose, in 2021, the samples were more resistant in Tengrela than Tiefora (Fig. 3A-D, right-hand density plots).

The fit of the models to the field data were notably worse than for the laboratory data (Supplementary file 1: Figures S4.1-S4.4 compared to Fig. S3 for the laboratory model assessment), as expected given the inherent variation in both experimental conditions and in the wild mosquito populations. The model shows reasonable and similar predictive accuracy in both Tengrela and Tiefora and residuals are balanced across the concentration range in both locations (Supplementary file 1: Fig. S4).

Generally, a slightly higher background mortality rate was estimated in Tiefora (around 1.6% across all models) than Tengrela (mean 1%; Supplementary file 1: Table S2).

## 4 Discussion

Here, we introduce a novel Bayesian framework for analysis of intensity bioassay data which can be used to quantify insecticide resistance in malaria vectors. Using this approach, we generated a series of quantitative measures of resistance for laboratory strains with well-studied resistance profiles and for field-derived specimens. These measures included estimates of the variation in lethal doses across surveyed mosquitoes, which may represent heterogeneity in the resistance level in the population. Our method is flexible and able to straightforwardly incorporate observation- or study-level covariates, and here we showcase this by estimating the changing shape of resistance profiles in two high resistance locations in Burkina Faso. Our framework could further be used to quantify insecticide resistance in other mosquito species or insects.

In toxicological or pharmacological studies, metrics such as the LC_50_ or the slope gradient are conventionally reported and have clear, defined meanings. Within the context of phenotypic resistance monitoring for public health, what these metrics represent epidemiologically is unclear. The LC_50_ represents the point of the curve at which the amount of relative error in determining the lethal concentration is minimised in comparison to other points along the curve (Trevan, 1927; Burgess et al., 2020). Furthermore, this value was shown here to clearly distinguish between susceptible and resistant mosquito strains within the range of observed data (Table 3), as well as show increases in the magnitude of resistance (Supplementary file 1: Table S2). Different LC values could be selected if relevant in a public health context by rearranging the model to show, for example, the percentage of mosquitoes which die following exposure to the concentration of insecticide bioavailable on insecticide treated nets (Supplementary file 1: Equations S3). Though this may be informative, care should be taken to not overly interpret the results as many parameters, for example exposure time in bioassays and free-flying mosquitoes, will be different. In a field context, some intensity bioassays never reach 100% mortality, in which case the LC_50_ is also a more reliable metric of comparison than higher LCs and this metric is easier to obtain with smaller test subjects (Trevan, 1927). However, it is worth noting that the LC_50_ is not relatable to either the current discriminating dose for different insecticides or the concentration used on different vector control tools, so it would rather be of use as a measure of resistance intensity over only reporting its prevalence (Dennehy et al., 1983; Roush and Miller, 1986; Lipnick et al., 1995; Kranthi, 2005; White et al., 2011).

The gradient of the dose-response curve is likely to be informative, with a flatter slope being indicative of more heterogeneity in resistance within a population (Trevan, 1927). The parameter value itself might not be intuitive in a field context, so here we present new ways of expressing this population heterogeneity, including the probability density of the lethal dose or the LC_10_-LC_90_ range. In the genetically defined inbred colony mosquito populations compared here, resistant strains exhibited slightly more heterogeneity than the susceptible strain (Fig. 2B). This may result from the maintenance of insecticide selection pressure over time since their colonisation. Field mosquitoes exhibited much higher heterogeneity overall than laboratory stains (Fig. 3B), which is to be expected with the more complex genetic background of field samples (Halliday and Burnhaw, 1990) and species composition variation throughout the collection season. The heterogeneity observed in the field also appeared to increase as resistance increases (Supplementary file 1: Table S2).

In the field data, comparing the *temporal* models to the *base model* for each field location (i.e. *fixed resistance model*), all time models (i.e. *resistance change models*) fitted the data significantly better in Tengrela (*P* ​< ​0.01) but not Tiefora (Supplementary file 1: Table S1). This provides the first evidence that we are aware of for a systematic change in the intensity of resistance in a wild mosquito population over time. Interestingly, this change over time was observed in Tengrela but not in Tiefora, despite being relatively similar geographically and both villages having high use of pyrethroid ITNs. However, it is worth noting that collections from Tengrela came from a single large rice field, whereas in Tiefora they came from multiple breeding sites. Overall, we saw consistently and significantly higher LC_50_ estimates in Tengrela compared to Tiefora (Supplementary file 1: Table S2). Though mosquitoes from Tengrela appear to have higher resistance, mosquitoes from Tiefora exhibited higher heterogeneity, with the LC_10_-LC_90_ range being on average 138× ​DC compared to 17.5× ​in Tengrela (Supplementary file 1: Table S2). Though the difference in the distribution of the lethal dose seems clear between the two villages, care should be taken interpreting the LC_10_-LC_90_ range from Tiefora as a substantial proportion of mosquitoes survived the highest dose tested in this village, so LC_90_ estimates had to be inferred from the shape of the chosen curve (i.e. reliant on chosen model priors). This highlights both the strengths of the framework, as values can be generated and comparisons made, but also a frailty, as these values can easily be overly interpreted. Ideally, to obtain a precise curve, the dose of insecticide in the IB experiments should capture a change from no mortality to 100% mortality, which could involve increasing (above 10× ​or 20× ​DC) or decreasing (below 1× ​DC). However, in practice this might not happen for logistical reasons, such as the number of mosquitoes collected. In such a situation it seems appropriate to highlight LC estimates beyond the range of the data to prevent misunderstanding. The exact cause of the higher heterogeneity observed in Tiefora is unknown, though it could be related to the collection methodology (high number of breeding sites, as mentioned above), different species within the *An. gambiae* (*s.l.*) complex, or species composition variation throughout the collection season.

The work also shows how the accuracy of the dose-response curves can be assessed and should be reported. In the laboratory strains, all datapoints were generally observed very close to the best-fit curve, on average 4.5% away. This is compared to field data where on average points were 9.6% away in Tengrela and 10.6% away in Tiefora. These phenotypic assays have been shown to have some inherent variability, amplified by field variables such as species composition, mosquito weight, season or habitat (Althoff and Huijben, 2022), so an increase in assay variability is obviously to be expected. The quantitative estimates of goodness-of-fit generated here can be used as a measure of reliability in IB results, potentially flagging when measurement error is high and might require the assay to be repeated to generate robust results.

The framework outlined here can take a range of different functional forms to capture the relationship between mosquito mortality and insecticide concentration. Here the five-parameter logistic function was selected over the more common Hill function (Stepniewska et al., 2007; Goutelle et al., 2008; Prinz, 2010) so that background mortality could be quantified and accounted for. To allow for a range of different curve profiles, a five-parameter logistic function was chosen over a four-parameter one as it has been shown to be more appropriate for real-life data (Cumberland et al., 2015; Althoff and Huijben, 2022) and allows the curve to be asymmetric (Gottschalk and Dunn, 2005; Liao and Liu, 2009). Other work modelling IB data have also shown that probit models could accurately model this type of data and its variance (Althoff and Huijben, 2022; Karunarathne et al., 2022). The main difference between these two modelling approaches is on the distribution of the error term: the probit model assumes normal distribution of the error term, whereas a logistic model assumes that it follows a logistic distribution (i.e. slightly longer tails). In one study comparing the two approaches, the logistic model showed a higher goodness-of-fit (Althoff and Huijben, 2022).

Ultimately, the best functional form to use will depend on the quality of the data and the exact question under investigation. To exemplify our Bayesian framework, priors were selected to be relatively uninformative to minimise bias and allow a wide variety of dose-response curves (Supplementary file 1: Fig. S2). In a real-life context where data might be more sparse, more informative priors would likely be appropriate.

Our framework is phenomenological in nature, since we use it to probe phenotypic resistance, where potentially a wide range of biological mechanisms may underpin the complex patterns of resistance we identified, particularly in field populations. For laboratory populations with known resistance mechanisms, it would be interesting to use a combination of experimentation and mathematical modelling to explore mechanistic explanations that underpin their dose-response curves. The results from these well-controlled systems could help to interpret field data from populations where particular resistance mechanisms are known to predominate. On average, the framework fitted both susceptible and resistant laboratory strains equally well (Supplementary file 1: Fig. S3A). As a result, more variability in the model was not an indicator of resistance (Table 3 and Supplementary file 1: Table S2, mortality variability column). Higher model variability also appears not to be linked to population heterogeneity (Fig. 2, Fig. 3, Table 3 and Supplementary file 1: Table S2). The mean absolute error was the metric chosen to represent model variability, whereas the root mean square error was used to assess the model fit. Both of these metrics represent average model error prediction; however, it is worth noting that the former is more sensitive to error variance and the latter gives a higher weight to outliers. Conscious of the limitation of each summary metric, the mean absolute error was used to determine the variability on account of both its interpretability and its sensitivity to variance.

In settings where resistance is present, a better understanding of that resistance will allow appropriate programmatic decisions to be made to limit the amount of selection pressure imposed on malaria vectors (Halliday and Burnhaw, 1990; Kranthi, 2005; Burgess et al., 2020). Whilst it is not feasible to directly correlate bioassay results to field efficacy (Halliday and Burnhaw, 1990; Venter et al., 2017), this framework brings value in providing a quantitative description of the resistance which could help with the interpretation of efficacy trials on insecticide-based vector control. It has shown value in describing dose-response relationships to determine insecticide discriminating concentrations in mosquitoes (Corbel et al., 2023) and could be extended to other insects.

## 5 Conclusions

The framework allows the generation of a suite of new metrics describing the intensity of insecticide resistance. The relationship between these metrics and the effectiveness of mosquito control remains unclear and needs to be investigated. The DDB has proved a useful tool for predicting sub-optimal responses to insecticides used in malaria control, but in the future more nuanced and sensitive assays will be needed to guide programmatic decision making. The IB data have the potential to provide substantially more information than the DDB though its increased complexity needs adoption of more rigorous method of analysis, such as this framework. This framework could be extended to describe dose-relationships in other insects, to other insecticides or to explore specific covariates of interest.

## Funding

This work was funded by the 10.13039/501100000265Medical Research Council 10.13039/501100000761Imperial College London Doctoral Training Partnership (MRC-DTP), the 10.13039/100010269Wellcome Trust (200222/Z/15/Z) MiRA, and the 10.13039/100000865Bill & Melinda Gates Foundation (under Grant Agreement No. INV-010445). M.D.K., B.L. and T.S.C. acknowledge funding from the 10.13039/501100000265MRC Centre for Global Infectious Disease Analysis (reference MR/R015600/1), jointly funded by the UK 10.13039/501100000265Medical Research Council (10.13039/501100000265MRC) and the UK Foreign, Commonwealth & Development Office (FCDO), under the 10.13039/501100000265MRC/FCDO Concordat agreement, the EDCTP2 programme supported by the 10.13039/501100000780European Union, and Community Jameel. Findings and conclusions contained within the present paper are those of the authors and do not necessarily reflect positions or policies of the Bill & Melinda Gates Foundation.

## Ethical approval

Not applicable.

## CRediT authorship contribution statement

**Mara D. Kont:** Conceptualization, Methodology, Software, Formal analysis, Investigation, Resources, Writing – original draft, Writing – review & editing, Funding acquisition. **Ben Lambert:** Supervision, Conceptualization, Methodology, Writing – review & editing. **Antoine Sanou:** Resources, Writing – review & editing. **Jessica Williams:** Resources, Writing – review & editing. **Hilary Ranson:** Resources, Writing – review & editing. **Geraldine M. Foster:** Writing – review & editing. **Rosemary S. Lees:** Resources, Writing – review & editing, All authors read and approved the final manuscript. **Thomas S. Churcher:** Supervision, Conceptualization, Methodology, Writing – review & editing, Funding acquisition. All authors read and approved the final manuscript.

## Declaration of competing interests

The authors declare that they have no known competing financial interests or personal relationships that could have appeared to influence the work reported in this paper.

## Data Availability

The data supporting the conclusions of this article are included within the article and Supplementary file 1. The laboratory dataset can be found in Williams et al. (2019) and the field dataset in Sanou et al. (2021). The model code has been made freely available on GitHub (https://github.com/marakont/IB_framework/).

## References

[bib1] Abbott W.S. (1925). A method of computing the effectiveness of an insecticide. J. Econ. Entomol..

[bib2] Adolfi A., Poulton B., Anthousi A., Macilwee S., Ranson H., Lycett G.J. (2019). Functional genetic validation of key genes conferring insecticide resistance in the major African malaria vector, *Anopheles gambiae*. Proc. Natl. Acad. Sci. USA.

[bib3] Althoff R.A., Huijben S. (2022). Comparison of the variability in mortality data generated by CDC bottle bioassay, WHO tube test, and topical application bioassay using *Aedes aegypti* mosquitoes. Parasites Vectors.

[bib4] Alyokhin A., Mota-Sanchez D., Baker M., Snyder W.E., Menasha S., Whalon M. (2015). The Red Queen in a potato field: Integrated pest management *versus* chemical dependency in Colorado potato beetle control. Pest Manag. Sci..

[bib5] Bagi J., Grisales N., Corkill R., Morgan J.C., N'Falé S., Brogdon W.G., Ranson H. (2015). When a discriminating dose assay is not enough: measuring the intensity of insecticide resistance in malaria vectors. Malar. J..

[bib6] Bhatt S., Weiss D.J., Cameron E., Bisanzio D., Mappin B., Dalrympe U. (2015). The effect of malaria control on *Plasmodium falciparum* in Africa between 2000 and 2015. Nature.

[bib7] Black W.C., Snell T.K., Saavedra-Rodriguez K., Kading R.C., Campbell C.L. (2021). From global to local - new insights into features of pyrethroid detoxification in vector mosquitoes. Insects.

[bib8] Burgess E.R., IV, King B.H., Geden C.J. (2020). Oral and topical insecticide response bioassays and associated statistical analyses used commonly in veterinary and medical entomology. J. Insect Sci..

[bib9] Choo L.E., Tang C.S., Pang F.Y., Ho S.H. (2000). Comparison of two bioassay methods for determining deltamethrin resistance in German cockroaches (Blattodea: Blattellidae). J. Econ. Entomol..

[bib10] Corbel V., Kont M.D., Ahumada M.L., Andréo L., Bayili B., Bayili K. (2023). A new WHO bottle bioassay method to assess the susceptibility of mosquito vectors to public health insecticides: Results from a WHO-coordinated multi-centre study. Parasites Vectors.

[bib11] Cumberland W.N., Fong Y., Yu X., Defawe O., Frahm N., De Rosa S. (2015). Nonlinear calibration model choice between the four and five-parameter logistic models. J. Biopharm. Stat..

[bib12] Dennehy T.J., Granett J., Leigh T.F. (1983). Relevance of slide-dip and residual bioassay comparisons to detection of resistance in spider mites. J. Econ. Entomol..

[bib13] Enayati A., Hanafi-Bojd A.A., Sedaghat M.M., Zaim M., Hemingway J. (2020). Evolution of insecticide resistance and its mechanisms in *Anopheles stephensi* in the WHO Eastern Mediterranean Region. Malar. J..

[bib14] Gabry J., Simpson D., Vehtari A., Betancourt M., Gelman A. (2019). Visualization in Bayesian workflow. J. Roy. Stat. Soc.: Ser. A.

[bib15] Githeko A.K., Adungo N.I., Karanja D.M., Hawley W.A., Vulule J.M., Seroney I.K. (1996). Some observations on the biting behavior of *Anopheles gambiae s.s, Anopheles arabiensis*, and *Anopheles funestus* and their implications for malaria control. Exp. Parasitol..

[bib16] Glunt K.D., Oliver S.V., Hunt R.H., Paaijmans K.P. (2018). The impact of temperature on insecticide toxicity against the malaria vectors *Anopheles arabiensis* and *Anopheles funestus*. Malar. J..

[bib17] Glunt K.D., Thomas M.B., Read A.F. (2011). The effects of age, exposure history and malaria infection on the susceptibility of *Anopheles* mosquitoes to low concentrations of pyrethroid. PLoS One.

[bib18] Gottschalk P.G., Dunn J.R. (2005). The five-parameter logistic: A characterization and comparison with the four-parameter logistic. Anal. Biochem..

[bib19] Goutelle S., Maurin M., Rougier F., Barbaut X., Bourguignon L., Ducher M., Maire P. (2008). The Hill equation: A review of its capabilities in pharmacological modelling. Fundam. Clin. Pharmacol..

[bib20] Halliday R.W., Burnhaw K.P. (1990). Choosing the optimal diagnostic dose for monitoring insecticide resistance. J. Econ. Entomol..

[bib21] Ingham V.A., Anthousi A., Douris V., Harding N.J., Lycett G., Morris M. (2020). A sensory appendage protein protects malaria vectors from pyrethroids. Nature.

[bib22] Ingham V.A., Wagstaff S., Ranson H. (2018). Transcriptomic meta-signatures identified in *Anopheles gambiae* populations reveal previously undetected insecticide resistance mechanisms. Nat. Commun..

[bib23] Jones C.M., Sanou A., Guelbeogo W.M., Sagnon N.F., Johnson P.C.D., Ranson H. (2012). Aging partially restores the efficacy of malaria vector control in insecticide-resistant populations of *Anopheles gambiae s.l*. from. Burkina Faso. Malar. J..

[bib24] Karunarathne P., Pocquet N., Labbé P., Milesi P. (2022). BioRssay: An R package for analyses of bioassays and probit graphs. Parasites Vectors.

[bib25] Kleinschmidt I., Bradley J., Knox T.B., Mnzava A.P., Kafy H.T., Mbogo C. (2018). Implications of insecticide resistance for malaria vector control with long-lasting insecticidal nets: A WHO-coordinated, prospective, international, observational cohort study. Lancet Infect. Dis..

[bib26] Kranthi K. (2005).

[bib27] Lambert B. (2018).

[bib28] Liao J.J., Liu R. (2009). Re-parameterization of five-parameter logistic function. J. Chemom..

[bib29] Lindsay S.W., Thomas M.B., Kleinschmidt I. (2021). Threats to the effectiveness of insecticide-treated bednets for malaria control: thinking beyond insecticide resistance. Lancet Global Health.

[bib30] Lipnick R.L., Cotruvo J.A., Hill R.N., Bruce R.D., Stitzel K.A., Walker A.P. (1995). Comparison of the up-and-down, conventional LD_50_, and fixed-dose acute toxicity procedures. Food Chem. Toxicol..

[bib31] Lissenden N., Kont M.D., Essandoh J., Ismail H.M., Churcher T.S., Lambert B. (2021). Review and meta-analysis of the evidence for choosing between specific pyrethroids for programmatic purposes. Insects.

[bib32] Oliver S.V., Brooke B.D. (2014). The effect of multiple blood-feeding on the longevity and insecticide resistant phenotype in the major malaria vector *Anopheles arabiensis* (Diptera: Culicidae). Parasites Vectors.

[bib33] Prinz H. (2010). Hill coefficients, dose-response curves and allosteric mechanisms. J. Chem. Biol..

[bib34] Quiñones M.L., Norris D.E., Conn J.E., Moreno M., Burkot T.R., Bugoro H. (2015). Insecticide resistance in areas under investigation by the International Centers of Excellence for Malaria Research: A challenge for malaria control and elimination. Am. J. Hyg. Trop. Med..

[bib35] Riveron J.M., Tchouakui M., Mugenzi L., Menze B.D., Chiang M.-C., Wondji C., Manguin S., Dev V. (2018). Towards malaria elimination - a leap forward.

[bib36] Roush R.T., Miller G.L. (1986). Considerations for design of insecticide resistance monitoring programs. J. Econ. Entomol..

[bib37] RStudio Team (2020). http://www.rstudio.com/.

[bib38] Sanou A., Nelli L., Guelbéogo W.M., Cissé F., Tapsoba M., Quédraogo P. (2021). Insecticide resistance and behavioural adaptation as a response to long-lasting insecticidal net deployment in malaria vectors in the Cascades region of Burkina Faso. Sci. Rep..

[bib39] Sherrard-Smith E., Winskill P., Hamlet A., Ngufor C., NʼGuessan R., Guelbeogo M.W. (2022). Optimising the deployment of vector control tools against malaria: A data-informed modelling study. Lancet Planet. Health.

[bib40] Sokhna C., Ndiath M.O., Rogier C. (2013). The changes in mosquito vector behaviour and the emerging resistance to insecticides will challenge the decline of malaria. Clin. Microbiol. Infect..

[bib41] Spillings B.L., Coetzee M., Koekemoer L.L., Brooke B.D. (2008). The effect of a single blood meal on the phenotypic expression of insecticide resistance in the major malaria vector *Anopheles funestus*. Malar. J..

[bib42] Stan Development Team (2019). Stan modeling language users guide and reference manual. https://mc-stan.org.

[bib43] Stan Development Team (2021). RStan: the R interface to Stan. R package. https://mc-stan.org/rstan/.

[bib44] Stepniewska K., Chotivanich K., Brockman A., Day N.P.J., White N.J. (2007). Overestimating resistance in field testing of malaria parasites: Simple methods for estimating high EC_50_ values using a Bayesian approach. Malar. J..

[bib45] Thomas M.B., Read A.F. (2016). The threat (or not) of insecticide resistance for malaria control. Proc. Natl. Acad. Sci. USA.

[bib46] Trevan J.W. (1927). The error of determination of toxicity. Proc. Roy. Soc. Lond. B.

[bib47] Vehtari A., Gabry J., Magnusson M., Yao Y., Bürkner P.-C., Paananen T. (2023). loo: Efficient leave-one-out cross-validation and WAIC for Bayesian models.. https://mc-stan.org/loo/.

[bib48] Vehtari A., Gelman A., Gabry J. (2017). Practical Bayesian model evaluation using leave-one-out cross-validation and WAIC. Stat. Comput..

[bib49] Vehtari A., Gelman A., Simpson D., Carpenter B., Bürkner P.-C. (2021). Rank-normalization, folding, and localization: An improved Ȓ for assessing convergence of MCMC (with discussion). Bayesian Anal.

[bib50] Venter N., Oliver S.V., Muleba M., Davies C., Hunt R.H., Koekemoer L. (2017). Benchmarking insecticide resistance intensity bioassays for *Anopheles* malaria vector species against resistance phenotypes of known epidemiological significance. Parasites Vectors.

[bib51] Weetman D., Wilding C.S., Neafsey D.E., Müller P., Ochomo E., Isaacs A.T. (2018). Candidate-gene based GWAS identifies reproducible DNA markers for metabolic pyrethroid resistance from standing genetic variation in East African *Anopheles gambiae*. Sci. Rep..

[bib52] White M.T., Griffin J.T., Churcher T.S., Ferguson N.M., Basáñez M.-G., Ghani A.C. (2011). Modelling the impact of vector control interventions on *Anopheles gambiae* population dynamics. Parasites Vectors.

[bib53] WHO (2012). https://apps.who.int/iris/handle/10665/44846.

[bib54] WHO (2016). https://apps.who.int/iris/handle/10665/250677.

[bib55] WHO (2019). https://www.who.int/publications/i/item/9789241565653.

[bib56] WHO (2021). https://www.who.int/publications/i/item/9789240015791.

[bib57] WHO (2022). https://www.who.int/publications/i/item/9789240051089.

[bib58] Williams J., Flood L., Praulins G., Ingham V.A., Morgan J., Lees R.S., Ranson H. (2019). Characterisation of *Anopheles* strains used for laboratory screening of new vector control products. Parasites Vectors.

[bib59] Zoh M.G., Bonneville J.-M., Tutagata J., Laporte F., Fodjo B.K., Mouhamadou C.S. (2021). Experimental evolution supports the potential of neonicotinoid-pyrethroid combination for managing insecticide resistance in malaria vectors. Sci. Rep..

