# Supplementary material for: Characterising the intensity of insecticide resistance: A novel framework for analysis of intensity bioassay data
Source: Curr Res Parasitol Vector Borne Dis. 2023 Jun 16;4:100125. doi: 10.1016/j.crpvbd.2023.100125 (PMC10338328; doi:10.1016/j.crpvbd.2023.100125)

# Supplementary Material

## **Figure S1. Effect of varying the parameters of the 5-parameter logistic function.**

The effect of varying parameters *A* to *E* (from Equation 2, as described in Table 2) are shown in panels *A* to *E*. In each panel, the arrow points towards the impact of increasing that parameter value on the curve, whilst keeping all other parameters constant.


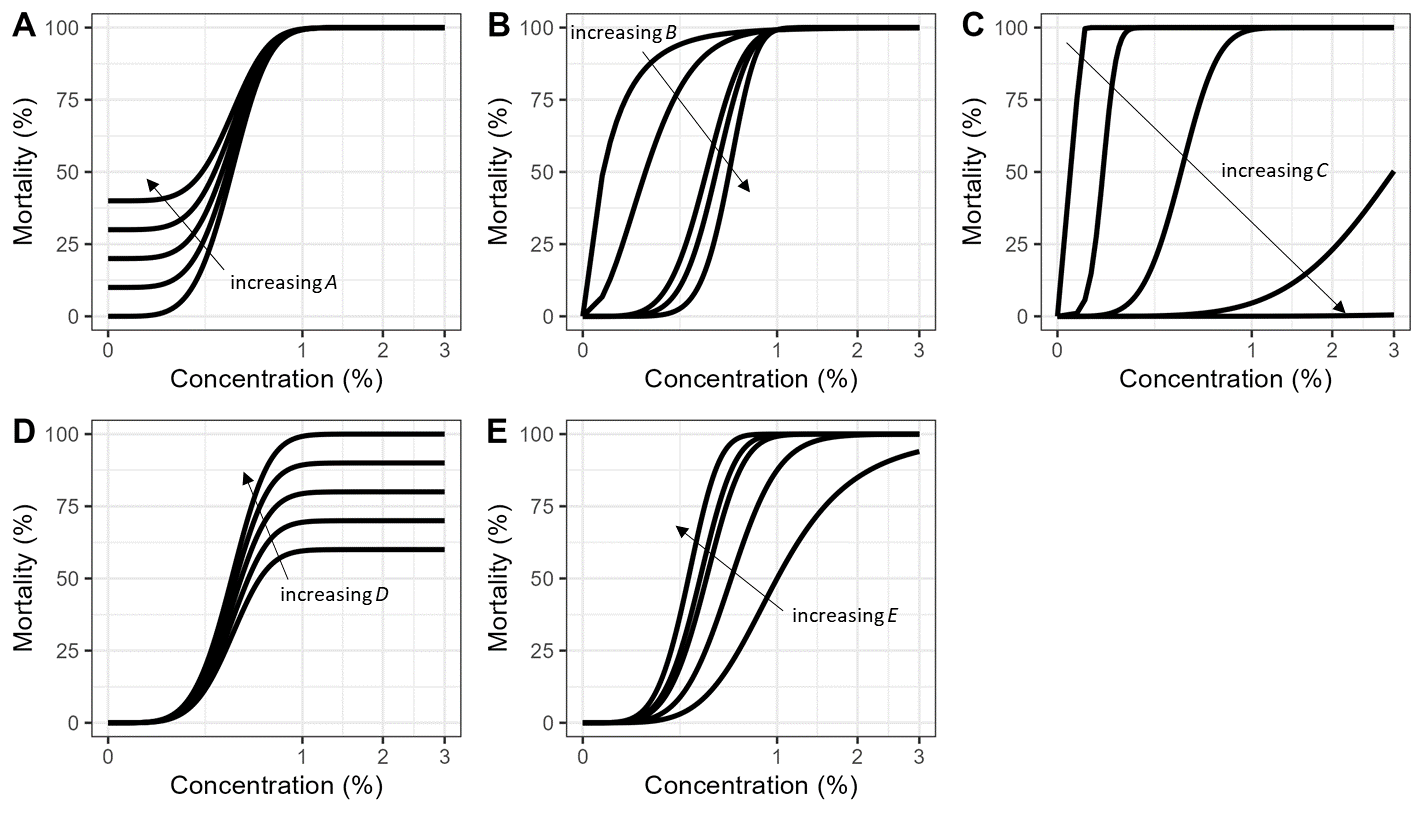


## **Figure S2. Base model prior predictive check**

Fixed model prior predictive check with priors for model detailed in Equation 2 specified as:
A ~ N(0, 0.1); *B* ~ N(5, 10); C ~ N(0, 5); and *E* ~ N(7, 10). Parameter *D* was fixed at a value of 1. The black line represents the median estimates of 1000 iterations and the shaded area represents the 95% confidence intervals with these prior settings.


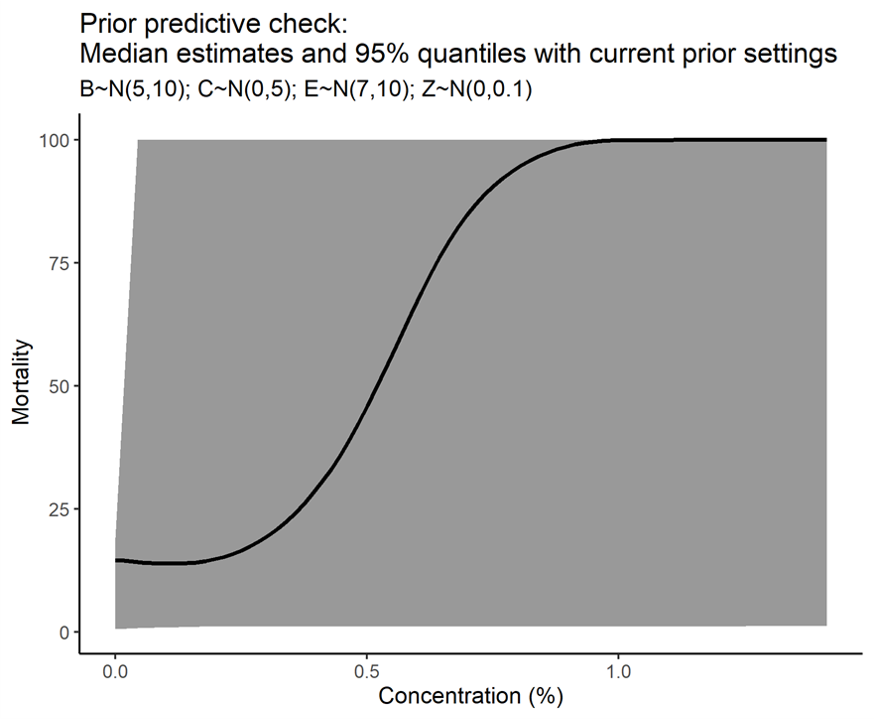


## **S3. Obtaining the LC_50_**

If $p_{i}=D+ \frac{A-D}{\left[ 1+e^{B\cdot\left( \ln\left( \sqrt{x_{i}} \right)-C \right)} \right]^{E}}$,

then:

${LC}_{x_{i}}=\left[ e^{\frac{ln\left( \left( \frac{A-D)}{{{(p}_{i}-D)}^{E}} \right)-1 \right)}{B}+C} \right]^{2}$.

## **Figure S3. Base model assessment**

*Base model* fitting assessment with A) actual versus predicted mortality values and respective *R^2^* and root mean square error (RMSE) values and B) model residuals per permethrin concentration, coloured by laboratory strain. In plot A, a perfect model prediction would result on all point lying on the dashed line, *R^2^*=1 and RMSE=0% and in plot B, all points would lie on the dashed line.


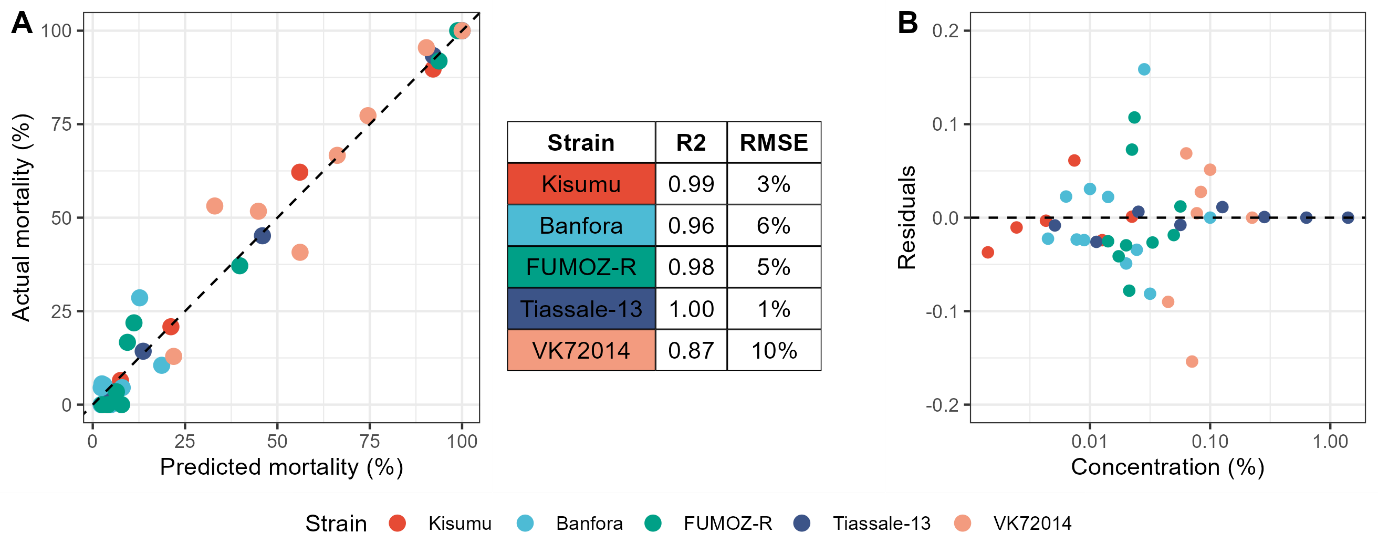


## **Table S1. Time models comparison**

Model comparison of the *time* models (Equations 3.1—3.3) using elpd estimates from the LOO-CV. Models are listed for each village with their respective equations in brackets. The best fitting model of the four is shown in italics and the first *p* value is obtained by comparing the best fitting model to each of the others. The second *p* value (last column) is obtained by comparing the fixed model (i.e. *fixed resistance*) to each of the time models (i.e. *resistance change* models).

| **Village** | **Model  (Equation)** | **elpd** | **SE** | **Best *vs* others** | **Fixed *vs* time** |
| --- | --- | --- | --- | --- | --- |
|  |  |  |  | ***p* value** | ***p* value** |
| Tengrela | *Base time (3.3)* | *-747.3* | *63.6* | *Comparator* | 0.003** |
|  | Individual time (3.2) | -749.8 | 61.4 | 0.45 | 0.002** |
|  | Linear time (3.1) | -785.6 | 60.0 | 0.14 | 0.002** |
|  | Fixed model (2) | -888.4 | 63.3 | 0.003** | *Comparator* |
| Tiefora | *Base time (3.3)* | *-1067.2* | *50.3* | *Comparator* | 0.11 |
|  | Individual time (3.2) | -1078.2 | 52.2 | 0.24 | 0.15 |
|  | Linear time (3.1) | -1085.8 | 54.1 | 0.18 | 0.14 |
|  | Fixed model (2) | -1096.3 | 56.6 | 0.11 | *Comparator* |

## **Table S2. Summary table and model estimates for the field data.**

For each field location, the number of raw data points and mosquitoes used to fit the time models is provided below. Mosquitoes in each location were exposed to increasing concentrations of deltamethrin in intensity bioassay over six consecutive years. Model estimates of the median lethal concentration (LC_50_) are generated by providing the mean of all iterations for each strain and 95% credible intervals (CIs). Mosquito population heterogeneity is investigated by examining the range of concentrations at which 10% and 90% of the mosquitoes die (mean LC_10_ and LC_90_­ of all model iterations for each location and year of data) and the difference between these two doses. The amount of background mortality is quantified from model estimates, with the mean of all model iterations for each location and year and 95% credible intervals provided below. The amount of variability in mortality is quantified from the average absolute distance of the data points to the best fit line along the mortality axis, with the median of all model iterations for each strain and 95% credible intervals provided below. These metrics are provided for each time model as follows:

## S2.1 Linear time model

Metrics described above are provided for the time model based on Equation (3.1) where parameter *C* varies in a linearly (positively or negatively) year on year whilst all other parameters are kept constant per location.

| **Linear time model** | | | | | | | | | | | | | | | |
| --- | --- | --- | --- | --- | --- | --- | --- | --- | --- | --- | --- | --- | --- | --- | --- |
| **Village** | **Year** | ***n* data points** | ***n* mosquitoes tested** | **Concentrations tested**  **(×DC deltamethrin)** | **LC_50_ (×DC deltamethrin)** | | **Heterogeneity (×DC deltamethrin)** | | | **Background mortality (% mortality)** | | | **Mortality variability (%)** | | |
|  |  |  |  |  | **Mean** | **95% CI** | **LC_10_** | **LC_90_** | **Diff** | | **Mean** | **95% CI** | | **Median** | **95% CI** |
| **Tengrela** | **2016** | 19 | 492 | 0 – 15 | 2.8 | 2.4 – 3.2 | 0.6 | 9.4 | 8.8 | | 0.6 | 0.3 – 0.9 | | 9.7 | 9.5 – 9.9 |
|  | **2017** | 74 | 1803 | 0 – 20 | 3.4 | 3 – 3.8 | 0.8 | 11.6 | 10.8 | |  |  |  |  |  |
|  | **2018** | 13 | 278 | 0 – 10 | 4.2 | 3.8 – 4.4 | 0.8 | 14.2 | 13.4 | |  |  |  |  |  |
|  | **2019** | 28 | 683 | 0 – 10 | 5.2 | 4.8 – 5.4 | 1 | 17.4 | 16.4 | |  |  |  |  |  |
|  | **2020** | 85 | 2098 | 0 – 10 | 6.4 | 6 – 6.8 | 1.4 | 21.6 | 20.2 | |  |  |  |  |  |
|  | **2021** | 56 | 1370 | 0 – 15 | 7.8 | 7.2 – 8.4 | 1.6 | 26.4 | 24.8 | |  |  |  |  |  |
| **Tiefora** | **2017** | 76 | 1972 | 0 – 20 | 3.8 | 3.4 – 4.2 | 0.8 | 156 | 155.2 | | 1.5 | 1.1 – 2 | | 11.1 | 11.0 – 11.2 |
|  | **2018** | 66 | 1601 | 0 – 20 | 3.6 | 3.2 – 3.8 | 0.8 | 143.8 | 143 | |  |  |  |  |  |
|  | **2019** | 27 | 600 | 0 – 10 | 3.2 | 3 – 3.6 | 0.8 | 132.8 | 132 | |  |  |  |  |  |
|  | **2020** | 113 | 2770 | 0 – 10 | 3 | 2.8 – 3.2 | 0.8 | 122.4 | 121.8 | |  |  |  |  |  |
|  | **2021** | 84 | 2030 | 0 – 15 | 2.8 | 2.6 – 3 | 0.6 | 113 | 112.4 | |  |  |  |  |  |

## S2.2 Individual time model

Metrics described above are provided for the time model based on Equation (3.2) where parameter C is allowed to vary independently per year whilst all other parameters are kept constant per location.

| **Individual time model** | | | | | | | | | | | | | | | |
| --- | --- | --- | --- | --- | --- | --- | --- | --- | --- | --- | --- | --- | --- | --- | --- |
| **Village** | **Year** | ***n* data points** | ***n* mosquitoes tested** | **Concentrations tested**  **(×DC deltamethrin)** | **LC_50_ (×DC deltamethrin)** | | **Heterogeneity (×DC deltamethrin)** | | | **Background mortality (% mortality)** | | | **Mortality variability (%)** | | |
|  |  |  |  |  | **Mean** | **95% CI** | **LC_10_** | **LC_90_** | **Diff** | | **Mean** | **95% CI** | | **Median** | **95% CI** |
| **Tengrela** | **2016** | 19 | 492 | 0 – 15 | 3 | 2.6 – 3.4 | 0.8 | 11.2 | 10.6 | | 0.7 | 0.4 – 1 | | 9 | 8.9 – 9.2 |
|  | **2017** | 74 | 1803 | 0 – 20 | 2.6 | 2.2 – 2.8 | 0.6 | 9.6 | 9 | |  |  |  |  |  |
|  | **2018** | 13 | 278 | 0 – 10 | 4.8 | 4 – 6 | 1.2 | 18.4 | 17.4 | |  |  |  |  |  |
|  | **2019** | 28 | 683 | 0 – 10 | 7.6 | 6.4 – 9.2 | 1.8 | 28.8 | 27 | |  |  |  |  |  |
|  | **2020** | 85 | 2098 | 0 – 10 | 7.4 | 6.8 – 8 | 1.8 | 28.2 | 26.4 | |  |  |  |  |  |
|  | **2021** | 56 | 1370 | 0 – 15 | 5.4 | 4.8 – 6 | 1.2 | 20.6 | 19.2 | |  |  |  |  |  |
| **Tiefora** | **2017** | 76 | 1972 | 0 – 20 | 3.4 | 3 – 3.8 | 0.6 | 120.8 | 120 | | 1.5 | 1.1 – 2 | | 10.9 | 10.9 – 11.0 |
|  | **2018** | 66 | 1601 | 0 – 20 | 3.8 | 3.2 – 4.8 | 0.8 | 135.6 | 134.8 | |  |  |  |  |  |
|  | **2019** | 27 | 600 | 0 – 10 | 5 | 3.8 – 6.4 | 1 | 172.2 | 171.2 | |  |  |  |  |  |
|  | **2020** | 113 | 2770 | 0 – 10 | 3.6 | 3.2 – 4.2 | 0.8 | 126 | 125.4 | |  |  |  |  |  |
|  | **2021** | 84 | 2030 | 0 – 15 | 2.4 | 2.2 – 2.8 | 0.4 | 84.4 | 83.8 | |  |  |  |  |  |

## S2.3. Base time model

Metrics described above are provided for the time model based on Equation (3.3) where all parameters are allowed to vary per year for each location.

| **Base time model** | | | | | | | | | | | | | | | | |
| --- | --- | --- | --- | --- | --- | --- | --- | --- | --- | --- | --- | --- | --- | --- | --- | --- |
| **Village** | **Year** | ***R*** | ***n* mosquitoes tested** | **Concentrations tested**  **(×DC deltamethrin)** | **LC_50_ (×DC deltamethrin)** | | **Heterogeneity (×DC deltamethrin)** | | | **Background mortality (% mortality)** | | | | **Mortality variability (%)** | | |
|  |  |  |  |  | **Mean** | **95% CI** | **LC_10_** | **LC_90_** | **Diff** | | **Mean** | **95% CI** | | | **Median** | **95% CI** |
| **Tengrela** | **2016** | 19 | 492 | 0 – 15 | 2.8 | 2.2 – 3.6 | 0.6 | 26.6 | 26 | | 3.1 | | 0.4 – 8.5 | | 8.5 | 8.3 – 8.7 |
|  | **2017** | 74 | 1803 | 0 – 20 | 2.6 | 2.4 – 3 | 0.4 | 10.2 | 9.8 | | 0.7 | | 0.1 – 1.6 | |  |  |
|  | **2018** | 13 | 278 | 0 – 10 | 5.2 | 3.8 – 7 | 0.6 | 34 | 33.4 | | 2.7 | | 0.4 – 7.3 | |  |  |
|  | **2019** | 28 | 683 | 0 – 10 | 7.6 | 6.6 – 9 | 2.2 | 18.8 | 16.8 | | 1.2 | | 0.3 – 2.9 | |  |  |
|  | **2020** | 85 | 2098 | 0 – 10 | 7.6 | 7.2 – 8.2 | 3 | 14.2 | 11.2 | | 1.8 | | 0.5 – 3.1 | |  |  |
|  | **2021** | 56 | 1370 | 0 – 15 | 5.8 | 5 – 6.4 | 1.6 | 14.6 | 13.2 | | 0.7 | | 0.2 – 1.6 | |  |  |
| **Tiefora** | **2017** | 76 | 1972 | 0 – 20 | 3.2 | 2.8 – 3.8 | 1 | 52.8 | 51.8 | | 1.4 | | 0.5 – 2.7 | | 10.8 | 10.6 – 10.9 |
|  | **2018** | 66 | 1601 | 0 – 20 | 4.4 | 3.6 – 5.2 | 0.6 | 560.4 | 559.8 | | 2.1 | | 0.9 – 3.6 | |  |  |
|  | **2019** | 27 | 600 | 0 – 10 | 5.8 | 4.8 – 6.8 | 1.2 | 17.2 | 16 | | 2.2 | | 0.6 – 4.9 | |  |  |
|  | **2020** | 113 | 2770 | 0 – 10 | 4.4 | 3.8 – 5 | 0.6 | 23.8 | 23.2 | | 1.4 | | 0.8 – 2.2 | |  |  |
|  | **2021** | 84 | 2030 | 0 – 15 | 3 | 2.2 – 3.8 | 0.2 | 120.6 | 120.4 | | 1.6 | | 0.8 – 2.7 | |  |  |

## **Figure S4. Time model assessment**

## S4.1 Fixed time model assessment

*Base model* (Equation 2) fitting assessment to field data with all years aggregated with A) actual versus predicted mortality values and respective *R^2^* and root mean square error (RMSE) values and B) model residuals per deltamethrin concentration. In plot A, a perfect model prediction would result on all point lying on the dashed line, *R^2^*=1 and RMSE=0% and in plot B all points would lie on the dashed line.


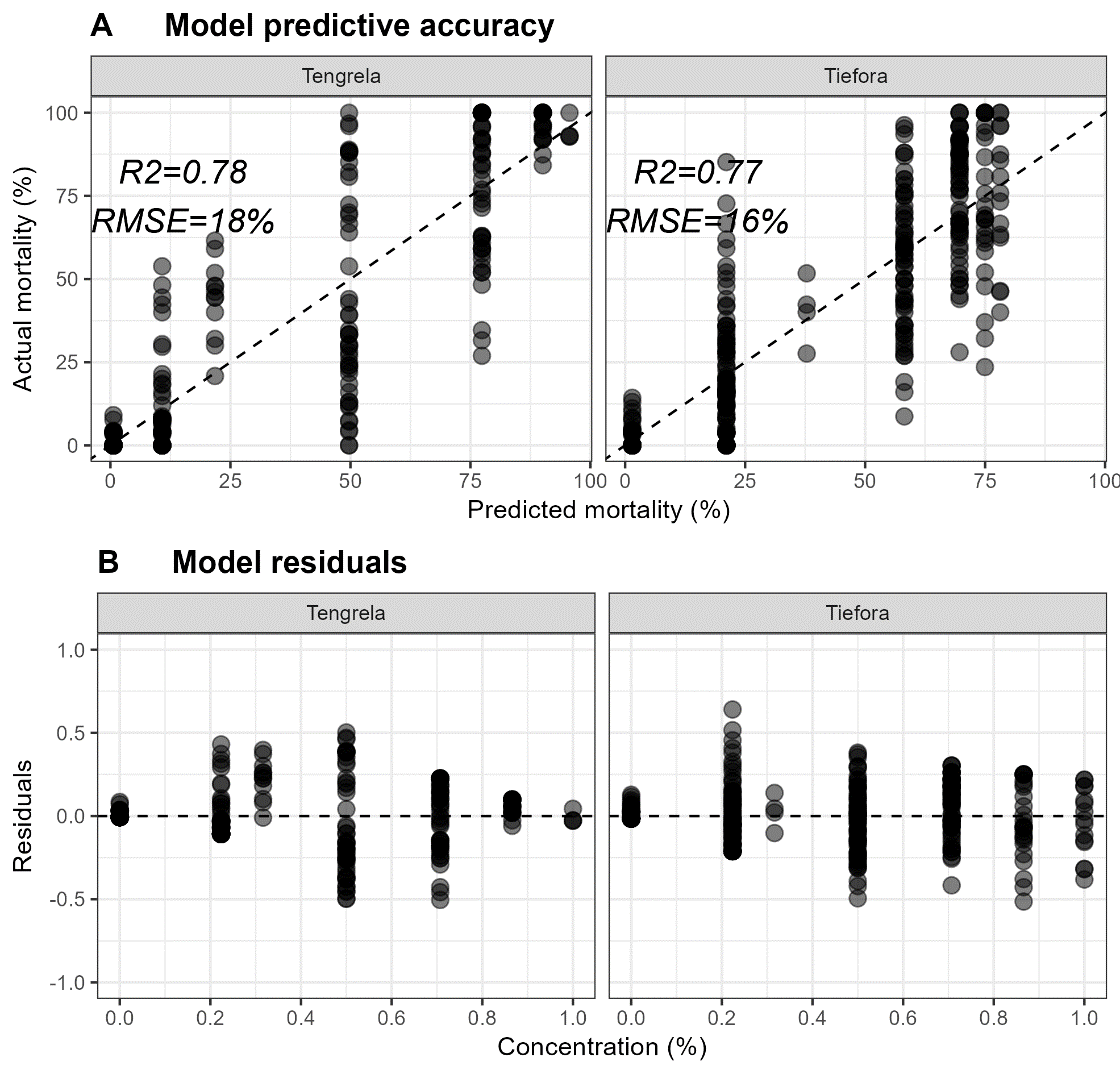


## S4.2 Linear time model assessment

*Linear time model* (Equation 3.1) fitting assessment with A) actual versus predicted mortality values and respective *R^2^* and root mean square error (RMSE) values and B) model residuals per deltamethrin concentration. In plot A, a perfect model prediction would result on all point lying on the dashed line, *R^2^*=1 and RMSE=0% and in plot B all points would lie on the dashed line.


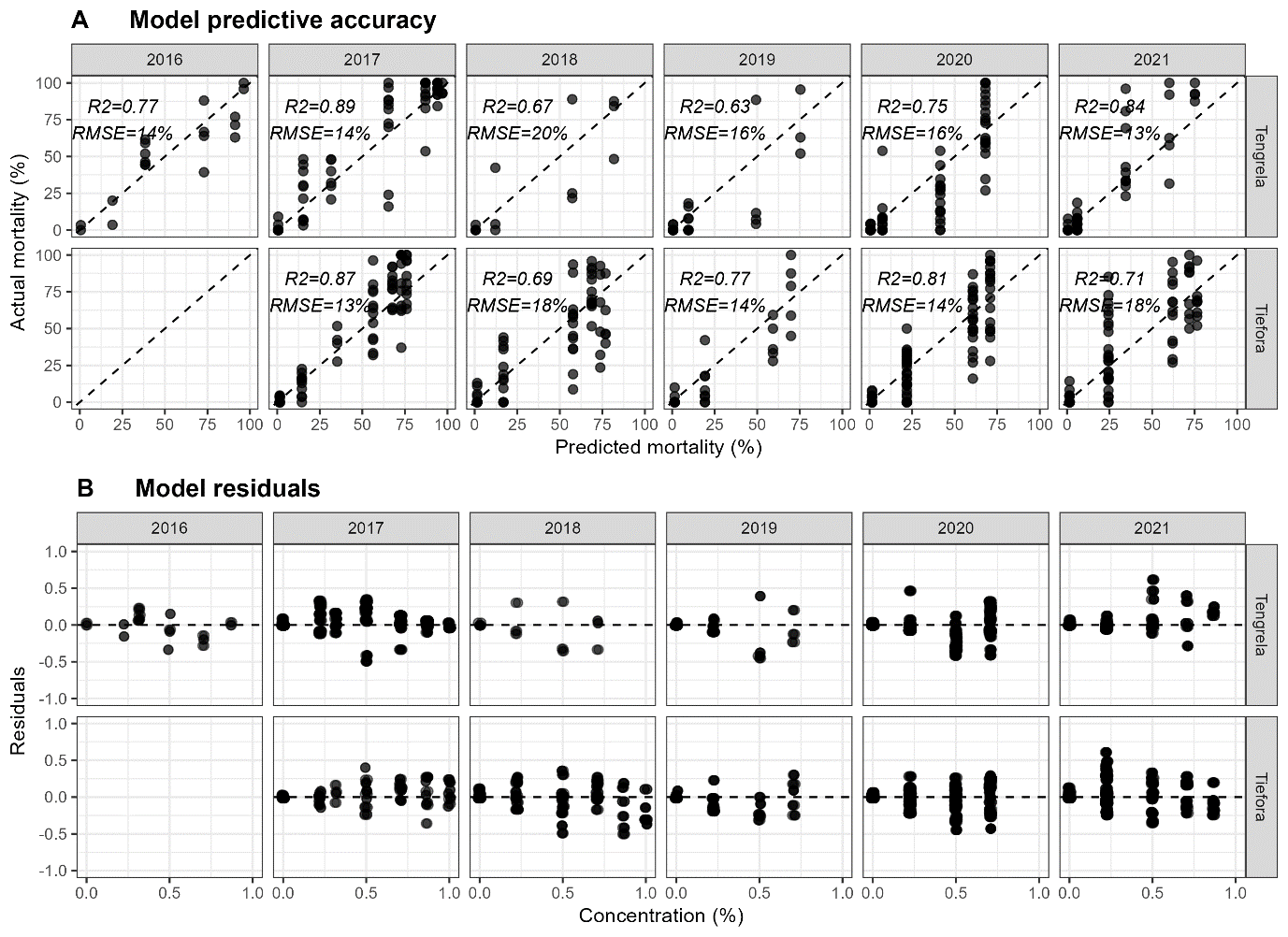


## S4.3 Individual time model assessment

*Individual time model* (Equation 3.2) fitting assessment with A) actual versus predicted mortality values and respective *R^2^* and root mean square error (RMSE) values and B) model residuals per deltamethrin concentration. In plot A, a perfect model prediction would result on all point lying on the dashed line, *R^2^*=1 and RMSE=0% and in plot B all points would lie on the dashed line.


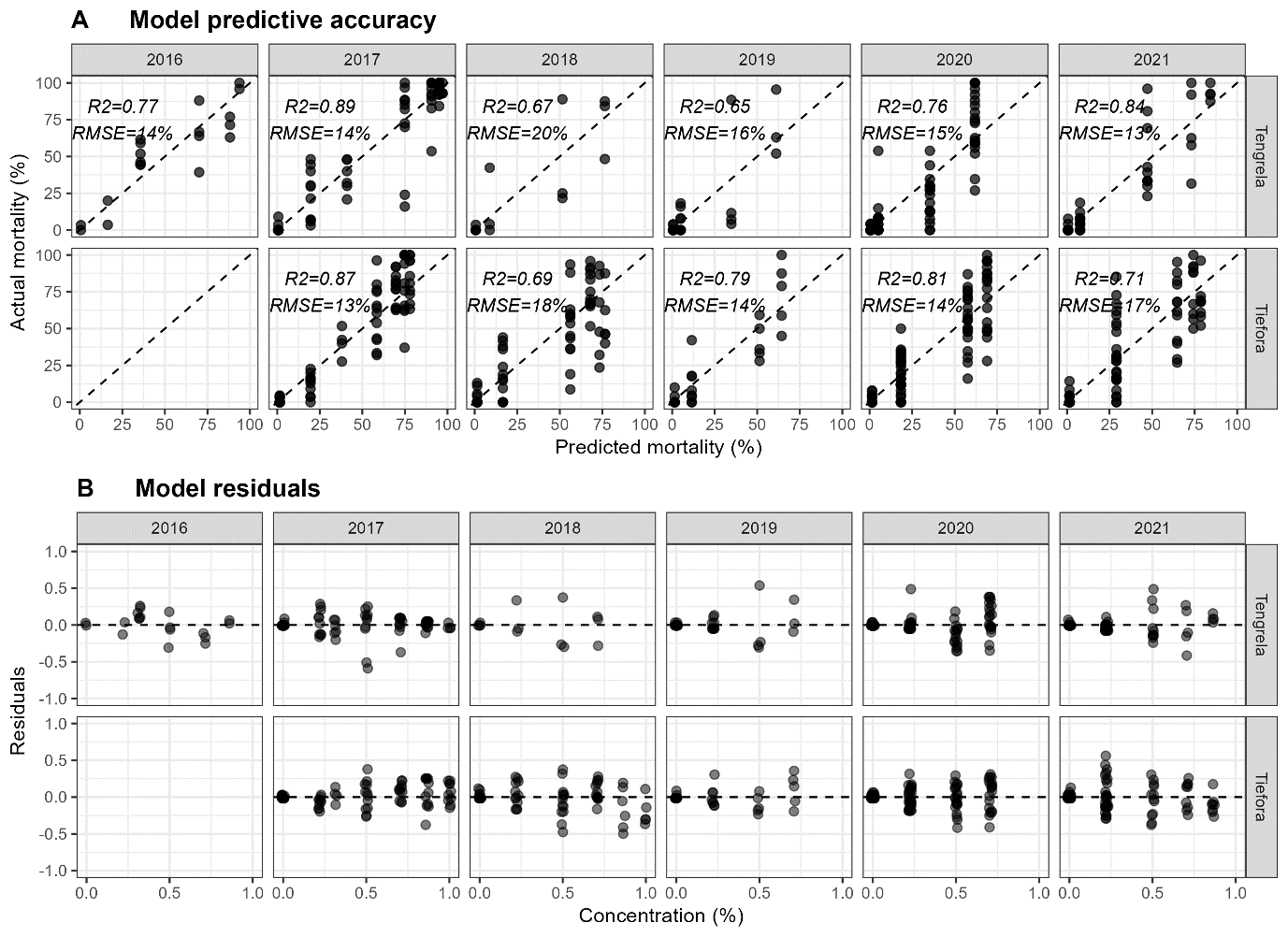


## S4.4 Base time model assessment

*Free time model* (Equation 3.3) fitting assessment with A) actual versus predicted mortality values and respective *R^2^* and root mean square error (RMSE) values and B) model residuals per deltamethrin concentration. In plot A, a perfect model prediction would result on all point lying on the dashed line, *R^2^*=1 and RMSE=0% and in plot B all points would lie on the dashed line.


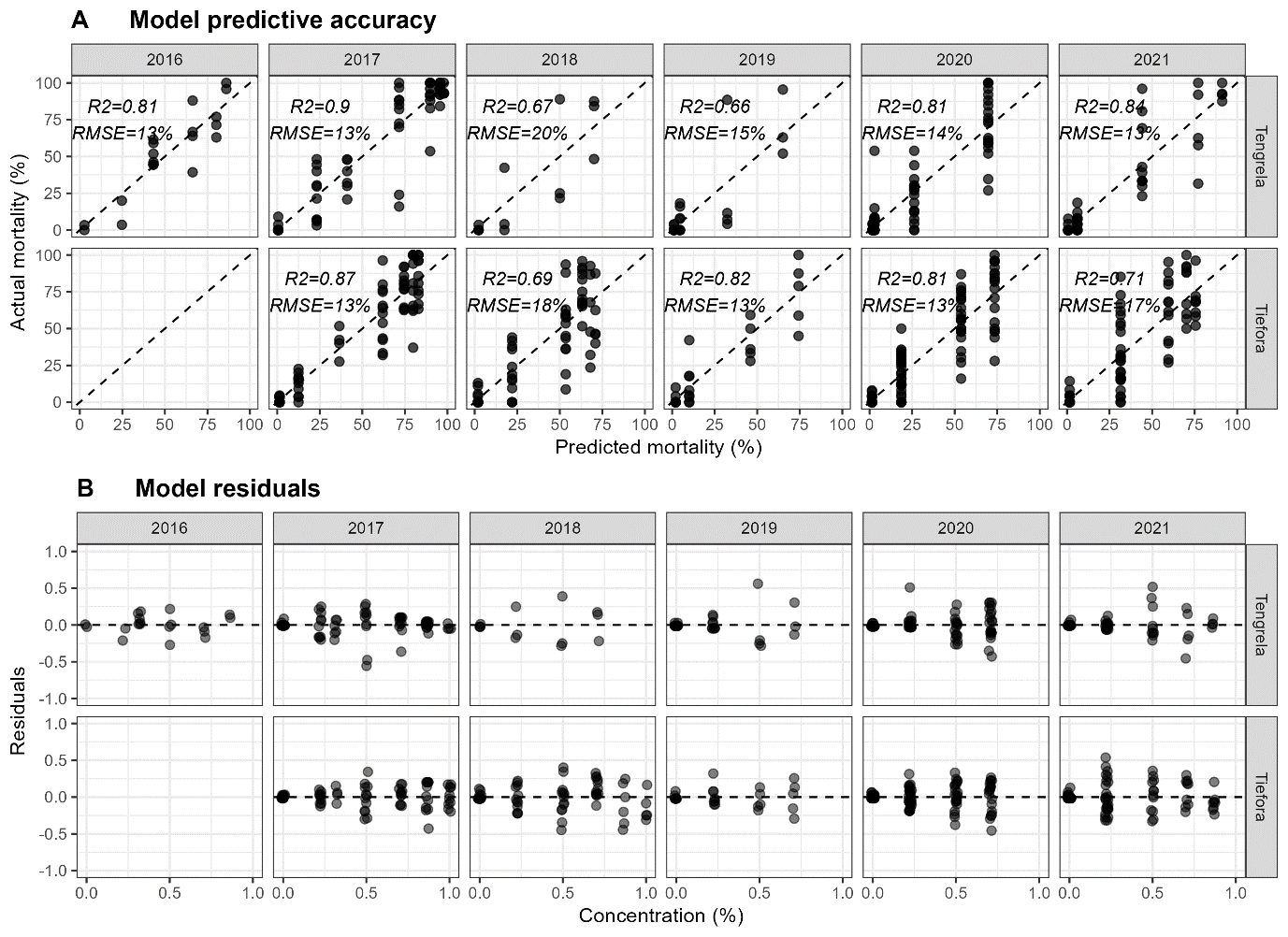

Supplement: Multimedia component 1 [file mmc1.docx]
